# Integrating multi-polygenic scores for enhanced prediction of antidepressant treatment outcomes in an East Asian population

**DOI:** 10.1038/s41386-025-02269-y

**Published:** 2025-10-28

**Authors:** Shu-Chin Lin, Chiu-Ping Fang, Chia-Lin Hsu, An-Nie Chung, Tzu-Ting Chen, Kai-Hsiang Hsu, Chueh-Chun Yeh, Jingyi Zheng, ChaoYu Liu, Chi-Shin Wu, Chia-Yen Chen, Po-Hsiu Kuo, Shih-Jen Tsai, Yu-Li Liu, Yen-Feng Lin

**Affiliations:** 1 Center for Neuropsychiatric Research, National Health Research Institutes, Miaoli, Taiwan; 2 Institute of Statistics and Data Science, National Taiwan University, Taipei, Taiwan; 3 Department of Psychiatry, Taipei City Psychiatric Center, Taipei City Hospital, Taipei, Taiwan; 4 Department of Mathematics and Statistics, Auburn University, Auburn, AL USA; 5 Department of Psychiatry, School of Medicine, Yale University, New Haven, CT USA; 6 National Center for Geriatrics and Welfare Research, National Health Research Institutes, Miaoli, Taiwan; 7 Department of Psychiatry, National Taiwan University Hospital Yunlin Branch, Yunlin, Taiwan; 8 Biogen, Cambridge, MA USA; 9 Department of Public Health & Institute of Epidemiology and Preventive Medicine, College of Public Health, National Taiwan University, Taipei, Taiwan; 10 Department of Psychiatry, Taipei Veterans General Hospital, Taipei, Taiwan; 11 Division of Psychiatry, School of Medicine, National Yang Ming Chiao Tung University, Taipei, Taiwan; 12 Department of Public Health & Medical Humanities, School of Medicine, National Yang Ming Chiao Tung University, Taipei, Taiwan; 13 Institute of Behavioral Medicine, College of Medicine, National Cheng Kung University, Tainan, Taiwan

**Keywords:** Depression, Predictive markers, Predictive markers, Genetics research

## Abstract

Major Depressive Disorder (MDD) significantly impacts global public health, yet the effectiveness of antidepressant treatments varies widely across individuals. This study addresses an important gap in the literature by examining how multi-polygenic scores (PGSs) can improve predictions of selective serotonin reuptake inhibitor (SSRI) treatment outcomes in an East Asian population—a region where pharmacogenomic studies have been limited. We analyzed two Taiwanese cohorts: the Taipei Veterans General Hospital cohort (VGHTP, *N *= 177) and the National Health Research Institutes cohort (NHRI, *N *= 245), all receiving SSRIs. PGSs for 108 traits potentially relevant to SSRI treatment outcomes were derived from large-scale genome-wide association studies using PRS-CS and PRS-CSx, incorporating data from multiple ancestries. We combined these PGSs with demographic and clinical variables (e.g., baseline severity of depression, medication dosage) and employed generalized linear mixed models with L1-penalization (glmmLasso), as well as machine and deep learning algorithms, to identify and evaluate predictors. Our results revealed several important PGS predictors, notably related to insomnia, multisite chronic pain, and higher levels of inflammatory biomarkers, which consistently correlated with lower treatment efficacy. While the ensemble model achieved a modest area under the curve (AUC) of 0.631 for predicting responders/non-responders, integrating early improvement in depressive symptoms considerably boosted predictive accuracy (AUC = 0.859) for identifying remitters/non-remitters by week 8. These findings underscore the value of multi-PGS approaches and highlight the necessity of expanding pharmacogenomic research to non-European populations. Future studies with larger, diverse cohorts and additional biomarkers may further advance individualized therapeutic strategies and alleviate the burden of depression worldwide.

## Introduction

Major depressive disorder (MDD) exerts a substantial burden on non-fatal health worldwide. In 2017, an estimated 149.5–178.9 million individuals experienced MDD, reflecting a notable increase in years lived with disability—by 32.1% from 1990 to 2007, and by an additional 12.6% from 2007 to 2017 [1]. Although antidepressants are generally regarded as first-line treatments for MDD, their outcomes remain suboptimal. Only about one-third of patients prescribed antidepressants achieve remission following their initial course of treatment [2], and more than one-third show resistance to sequential antidepressant interventions [3, 4].

A variety of demographic and clinical factors have been investigated to explain the variability in antidepressant responses. Several studies have associated greater baseline severity of depression with poorer treatment outcomes [5, 6], whereas Kilts et al. [7] reported differing correlations between baseline symptom severity and treatment efficacy depending on the specific antidepressant. While most studies have found no significant association between age and antidepressant outcomes [8–10], others suggest age-related variations in treatment efficacy [11, 12]. Likewise, although many studies report no significant gender-based differences [13–15], some have noted drug-specific gender effects [2, 16]. Additional factors, including socioeconomic status, substance use, brain imaging biomarkers, and personality disorders, have also been explored. However, despite these extensive efforts, no definitive conclusions have emerged regarding reliable demographic or clinical predictors of antidepressant treatment outcomes [17, 18].

Recent advances in pharmacogenetics highlight genetic factors that may influence the efficacy of antidepressants, including candidate genes and genetic variants [19–24]. Genome-wide association studies (GWAS) have further underscored a heritable component in remission following antidepressant treatment, with an estimated SNP-based heritability of *h*^*2*^ = 0.132 [24]. Of particular note are polygenic scores (PGS), which condense the genetic risk for specific traits into a single value and have shown considerable potential as biomarkers of medication response [25–27]. Nonetheless, the predictive accuracy of PGS directly related to antidepressant outcomes can be hampered by relatively small GWAS sample sizes (for instance, the study by Pain et al. [24] on antidepressant remission involved 5218 individuals). In light of these limitations, multiple studies have turned to PGSs for traits linked to MDD or those influencing antidepressant pharmacokinetics. For example, Fanelli et al. [28, 29] found that higher PGSs for MDD and schizophrenia correlated with poorer antidepressant responses, while Amare et al. [30, 31] identified associations between PGSs for personality traits, obesity, and coronary artery disease and response to selective serotonin reuptake inhibitors (SSRIs). Marshe et al. [32] further revealed that PGSs for cerebrovascular disease were related to antidepressant efficacy in late life. Moreover, recent research [33, 34] suggests that multi-polygenic scores (multi-PGS) can strengthen prediction accuracy for psychiatric disorders, a discovery that encourages the exploration of multi-PGS approaches for forecasting antidepressant outcomes.

While these studies demonstrate the predictive value of PGSs for antidepressant response, it is vital to recognize that the majority were conducted in European populations. Given that genetic ancestry can influence antidepressant outcomes [35, 36], extending this line of research to non-European populations is crucial. However, the limited availability of GWAS data presents an obstacle for such investigations. To date, no research has systematically examined PGS-based predictions of antidepressant treatment outcomes in East Asian populations.

To address this gap, our study utilized data from two Taiwanese clinical cohorts receiving SSRI therapy for MDD: the Taipei Veterans General Hospital cohort (VGHTP, *N *= 177) and the National Health Research Institutes cohort (NHRI, *N *= 245). We generated PGSs using PRS-CS [37] and PRS-CSx [38], the latter leveraging cross-population GWAS summary statistics to improve PGS accuracy in non-European samples. Our analyses integrated conventional clinical characteristics with PGSs corresponding to 108 distinct traits and incorporated multiple analytical strategies to enhance predictive power and support variable selection. Specifically, we conducted single-trait correlation analyses, multi-PGS analyses using generalized linear mixed models with L1-penalization, and machine/deep learning methods. These findings not only pinpoint critical predictors of SSRI efficacy but also open up avenues for future research into the biological mechanisms and potential clinical applications related to SSRI treatment outcomes.

## Method

### Study sample

This study involves two distinct Taiwanese cohorts. The first cohort, referred to as the Taipei Veterans General Hospital cohort (VGHTP), consists of 177 patients recruited from the Taipei Veterans General Hospital [39–41]. The second cohort, termed the National Health Research Institutes cohort (NHRI), comprises 245 patients recruited from five hospitals in collaboration with the National Health Research Institutes [42–44]. In both cohorts, patients received thorough diagnostic evaluations by board-certified psychiatrists following DSM-IV criteria. This study was conducted in compliance with the Declaration of Helsinki and received approval from the Institutional Review Board of Taipei Veterans General Hospital (VGHIRB No.: 2014-06-001B). Written informed consent was obtained from each participant, ensuring that they fully understood the purpose and procedures of the research.

In the VGHTP cohort, fluoxetine, citalopram, and sertraline were prescribed. However, three patients on sertraline were excluded from the analyses due to their limited representation, leaving 174 VGHTP samples for further study. By contrast, the NHRI cohort received escitalopram or paroxetine. Treatment outcomes were evaluated based on the total score of the 17-item Hamilton Rating Scale for Depression (HRSD-17) across multiple follow-up assessments: weeks 0 (baseline), 4, and 8 for the VGHTP cohort, and weeks 0 (baseline), 1, 2, 4, 6, and 8 for the NHRI cohort.

All genotypic data underwent stringent quality control (QC), leading to the exclusion of four VGHTP patients during the QC process. Imputation was conducted with the Michigan Imputation Server, using the 1000 Genomes Phase 3 reference panels to harmonize genotypic data. Any single-nucleotide polymorphisms (SNPs) with a minor allele frequency below 0.01 or poor imputation quality (dosage R^2^ < 0.5) were removed. After these steps, approximately eight million imputed SNPs remained for the calculation of polygenic scores.

### Statistical Methods

#### Demographic statistics

We analyzed demographic characteristics and clinical factors that were common to both cohorts, including sex, age, number of depressive episodes, SSRI type and dosage, as well as baseline and follow-up HRSD-17 scores. SSRI dosage was quantified using the Defined Daily Dose (DDD) metric. Table 1 presents summary statistics for these variables in each cohort separately and in the pooled dataset. To evaluate potential differences between cohorts, we employed Pearson’s Chi-squared test for categorical data and the Wilcoxon-Mann-Whitney test for continuous data.

**Table 1:** Demographic statistics.

	Pooled data (*N *= 419)	VGHTP (*N *= 174)	NHRI (*N *= 245)	*P-*values^a^
Sex				3.3e−09
Male	29.2%	45.3%	18.0%	
Female	70.8%	54.7%	82.0%	
Drug				--
Fluoxetine	19.3%	47.1%	--	
Citalopram	21.7%	52.9%	--	
Paroxetine	37.1%	--	62.9%	
Escitalopram	21.9%	--	37.1%	
Age	43.7 (31.5–55.5)	46 (34–59)	41.3 (29.5–52.4)	0.0001
Baseline HRSD-17 score	24 (20–27)	26 (24–29)	21 (18–24)	5.7e−28
Number of depressive episodes	1 (1–2)	1 (1–1)	1 (1–2)	0.0012
Dose of antidepressant drug	1(0.75–1)	1 (0.75–1)	1 (1–1)	0.0284
P.I. at week 4	45.8 (28.9–60.7)	50 (34.7–60.8)	42.9 (23.8–60)	0.0038
Responders at week 4^b^				0.0342
Responders	44.1%	50.6%	39.6%	
Non-responders	55.9%	49.4%	60.4%	
P.I. at week 8	58.3 (40.9–73.5)	55.3 (43.0–72)	61.1 (36.8–75)	0.7734
Remitters at week 8^c^				0.0003
Remitters	35.7%	25.3%	43.3%	
Non-remitters	64.3%	74.7%	56.7%	

Demographic statistics are provided in percentages (%) for categorical variables and median (interquartile range) for continuous variables. The quantification of antidepressant dosage was determined using the Defined Daily Dose (DDD) metric.

*P.I.* percentage improvement in HRSD-17 scores.

^a^Pearson’s Chi-squared test and Wilcoxon–Mann–Whitney test are applied to test differences between the two cohorts for categorical data and continuous data, respectively.

^b^Responders are defined as patients with P.I. at week 4 $\ge$50%.

^c^Remitters are defined as patients with HRSD-17 score ≤7 at week 8.

#### PGS calculation

We collected approximately 200 GWAS summary statistics, primarily from East Asian and European populations, to compute polygenic scores (PGSs) for 108 traits relevant to MDD or implicated in SSRI pharmacokinetics. These traits span various domains, including psychiatric conditions (e.g., bipolar disorder, obsessive-compulsive disorder), substance use (e.g., cigarettes per day), personality and behavioral traits (e.g., neuroticism), brain biomarkers and neurological disorders (e.g., cortical thickness, Alzheimer’s disease), sleep traits (e.g., insomnia, sleep duration), inflammation biomarkers (e.g., C-reactive protein), medical diseases (e.g., stroke), and medical measurements (e.g., body mass index, diastolic blood pressure). Comprehensive details of these 108 traits and their corresponding GWAS references can be found in the Supplementary Table S2.

To generate polygenic scores, we used two advanced methods: PRS-CS [37] and PRS-CSx [38]. PRS-CS was applied for traits corresponding to a single GWAS. In cases where multiple GWAS of the same ancestry existed, we utilized METAL [45] to generate meta-GWAS summary statistics, which were then used to calculate PGSs. For instance, GWAS data on body fat rate were obtained from both the Taiwan Biobank (https://www.twbiobank.org.tw/) and Biobank Japan [46, 47], both representing East Asian populations. We first integrated these data into an East Asian meta-GWAS using METAL, then calculated PGS based on the resulting summary statistics. For traits with GWAS from multiple ancestries, we employed PRS-CSx—a Bayesian approach designed to enhance the predictive power of PGS by leveraging large cross-population GWAS data. Both PRS-CS and PRS-CSx utilized the 1000 Genomes reference panels under default parameters. For PRS-CSx, we employed the “meta” option to determine posterior SNP effect sizes across populations. We then generated PGSs for our study population using the “--score” command in PLINK 1.9 [48], applying the SNP effect sizes produced by PRS-CS and PRS-CSx. To address potential population stratification, the PGSs were regressed on the first 10 genotype principal components (PCs), which were derived from a set of independent SNPs (pruning SNPs with minor allele frequency >0.05 using a window size of 200 kb and r^2^ < 0.1) using PLINK. We extracted the standardized residuals from these regressions for use as PGS predictors in subsequent analyses.

#### Outcome definitions

Three outcomes were considered in our analyses, including percentage improvement (P.I.) in HRSD-17 scores at week 4, response (defined as a 50% or more improvement in HRSD-17 score) at week 4, and remission (defined as having a HRSD-17 score ≤7) at week 8. While Pain et al. [24] showed negligible heritability for percentage improvement in a highly heterogeneous meta-analysis, we hypothesized that a harmonized East Asian cohort might reveal stronger genetic contributions. Moreover, modeling continuous symptom change complements binary response and remission measures that may overlook sub-threshold variability. We considered both response at week 4 and remission at week 8 as endpoints.

#### Pearson’s correlation

As an initial step, we computed Pearson’s correlations between each individual PGS predictor and the percentage improvement (P.I.) in HRSD-17 scores at week 4, considering both cohort-specific and pooled datasets. Detailed correlation results are provided in the Supplementary Table S3.

#### Linear mixed model and generalized linear mixed models with L1-penalization

To investigate how demographic and clinical factors influence the percentage improvement in HRSD-17 scores at various time points, we used a linear mixed model of the form:

$$P.I. \sim \,	{week}+{sex}+{age}+{age}\times {sex}+{number\; of\; depressive\; episodes}\\ 	 +{drug}+{dosage\; of\; SSRI\; drug}+{HRSD}-17{baseline\; score}+(1|{subject}),$$

where (1|subject) denotes random effects at the individual level, and age × sex indicates an interaction term between age and sex. This model was implemented using the “lmer” function from the “lmerTest” R package [49]. Results are presented in Supplementary Table S1 in Supplement Appendix 1.

We then extended this model by incorporating all PGS predictors derived from the 108 traits, leading to the following formulation:

$$P.I. \sim \,	{week}+{sex}+{age}+{age}\times {sex}+{number\; of\; depressive\; episodes}\\ 	 +{drug}+{dosage\; of\; SSRI\; drug}+{HRSD}-17{baseline\; score}\\ 	 +(1|{subject})+108{PGS\; predictors}$$

For this extended model, we used the “glmmLasso” R package [50] to apply generalized linear mixed models with L1-penalization [51]. Tuning parameters for the L1-penalty were chosen according to the Bayesian Information Criterion (BIC). The final selected model and its parameter estimates are detailed in Supplementary Table S4. All statistical analyses were carried out using R Statistical Software version 4.2.2 (https://www.r-project.org/).

#### Machine/deep learning algorithms

For our predictive modeling, we employed machine and deep learning techniques to classify responders versus non-responders at week 4 and remitters versus non-remitters at week 8. The model was trained on the NHRI dataset, and external validation was performed using the VGHTP dataset as a testing set.

We implemented several algorithms—L1-regularized logistic regression, support vector machine (SVM), and extreme gradient boosting (XGBoost)—using the Scikit-learn library version 1.0.2 [52]. For deep learning, we constructed a deep neural network (DNN) in Keras on TensorFlow 2.11.0 [53]. We further developed an ensemble model via the voting classifier in Scikit-learn, integrating outputs from various classifiers to bolster predictive accuracy.

A comprehensive grid-search strategy was employed to optimize algorithm hyperparameters, aiming to maximize cross-validation AUC. This was assessed through 10 iterations of repeated, 5-fold stratified cross-validation on the training dataset. We then evaluated the best model’s performance on the testing set, reporting the AUC alongside a 95% confidence interval generated via 1000 bootstrap replicates. Supplementary Figs. S3 and S4 in Supplement Appendix 2 provide the workflow of the aforementioned analysis, more information regarding model configurations, and the grid search process. For additional interpretability, we used Shapley additive explanation (SHAP) [54] to identify the most influential variables in the top-performing model. All relevant analyses were implemented in Python 3.9.12.

#### Adjusted R-squared analysis

To determine the contribution of multi-PGS predictors to explaining SSRI outcomes, we computed adjusted R-squared (R^2^) values for linear models. Here, the outcome variable was P.I. at week 4, and the analyses were conducted on the pooled dataset with various combinations of multi-PGS predictors. The baseline linear regression model, incorporating only demographic and clinical factors, was:

$$P.I.{at\; week}4 \sim \,	{sex}+{age}+{age}\times {sex}+{number\; of\; depressive\; episodes}\\ 	 +{drug}+{dosage\; of\; SSRI\; drug}+{HRSD}-17{baseline\; score}.$$

Subsequently, we calculated adjusted R^2^ by adding PGS predictors identified through different selection strategies, including 1) PGSs significantly correlated with P.I. at week 4 (corPGS), 2) PGSs chosen by glmmLasso (lassoPGS), 3) PGSs among the top 10 features selected by SHAP values (mlPGS), and 4) the union of these PGS sets (unionPGS). For completeness, we also measured adjusted R^2^ using all 108 PGSs (wholePGS).

## Results

Table 1 presents a summary of demographic and clinical data for both cohorts, as well as the pooled analysis. We observed significant differences between cohorts in several demographic and clinical variables—such as sex, age, and baseline HRSD-17 scores. In view of these variations, we conducted both a pooled analysis and separate cohort-specific analyses of the estimated associations.

We initially applied Pearson’s correlation in the pooled dataset to evaluate potential relationships between the P.I. at week 4 and individual PGS predictors. Nominal significance (*P* < 0.05) was observed for negative correlations with panic disorder, insomnia, interleukin-16, multisite chronic pain, and heart rate, whereas a positive correlation was noted for the religious group. These findings were consistent across cohort-specific analyses. However, none of the PGS predictors passed the multiple testing correction threshold based on the false discovery rate [55], as shown in the Supplementary Table S3.

For the multi-PGS analysis, we used glmmLasso for variable selection. In the pooled analysis, the final model showed significant associations between PGS predictors and the P.I. Specifically, extraversion, insomnia, interleukin-12p70, interleukin-16, multisite chronic pain, panic disorder, and total bilirubin were negatively correlated with the P.I., while hip circumference, ischemic stroke, and stroke were positively correlated. These results are summarized in Table 2, with more detailed effect sizes and cohort-specific outcomes provided in the Supplementary Table S4.

**Table 2:** Summary of selected polygenic score (PGS) predictors based on three criteria.

r with P.I. at week 4	glmmLasso selection	SHAP-value selection
Panic disorder (−) **Insomnia (−)** Interleukin-16 (−) **Multisite chronic pain (−)** Heart rate (−) Religious group (+)	Extraversion (−) Hip circumference (+) **Insomnia (−)** Interleukin-12p70 (−) Interleukin-16 (−) Ischemic stroke (+) **Multisite chronic pain (−)** Panic disorder (−) Stroke (+) Total bilirubin (−)	Interleukin-13 **Multisite chronic pain** **Insomnia** Diastolic blood pressure Heart rate Autism spectrum disorder Platelet

Pearson’s correlation (r with *P*-value <0.05) in pooled analysis, glmmLasso-selected variables in pooled analysis, and SHAP-value-selected variables (computed through the best model in predicting responders: Ensemble). Bold text indicates the PGS predictors selected by all criteria.

(−) indicates a negative correlation or effect. (+) indicates a positive correlation or effect.

*P.I.* percentage improvement in HRSD-17 scores.

**Fig. 1:**
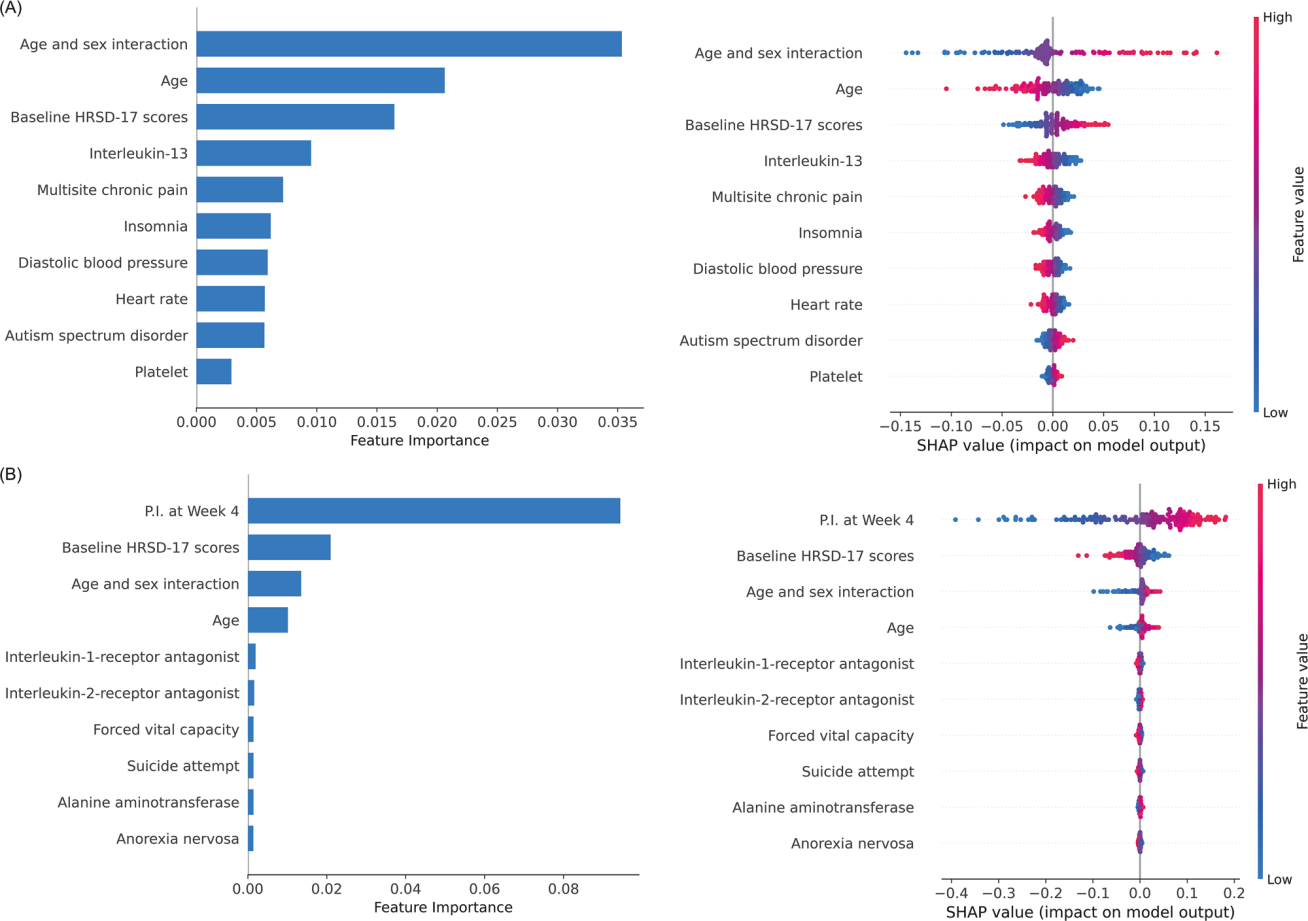
Feature importance and SHAP summary beeswarm plots for the best models. **A** Feature importance plot (left) and SHAP beeswarm summary plot (right) for the Ensemble model, displaying the top 10 important features and their SHAP values in predicting responders. **B** Feature importance plot (left) and SHAP beeswarm summary plot (right) for the SVM model, displaying the top 10 important features and their SHAP values in predicting remitters. (P.I. percentage improvement in HRSD-17 scores).

Table 3 compares the AUC values of the different machine and deep learning algorithms, along with their corresponding 95% confidence intervals. The ensemble model achieved the highest AUC, at 0.631 (95% CI: 0.544, 0.710), for distinguishing responders from non-responders. Figure 1A displays the top 10 predictors based on SHAP values. These predictors include key demographic factors—sex, age, and their interaction—consistent with linear mixed model findings (see Supplementary Table S1 and Supplementary Fig. S1 in Supplement Appendix 1). They also include PGS predictors for interleukin-13, multisite chronic pain, insomnia, diastolic blood pressure, heart rate, autism spectrum disorder, and platelet. Notably, Table 2 highlights the recurring selection of the PGSs for insomnia, multisite chronic pain, and inflammatory biomarkers across multiple approaches. In the SHAP beeswarm summary plot, higher PGS levels for insomnia and multisite chronic pain corresponded to lower SHAP values, indicating suboptimal SSRI responses—an observation in line with both Pearson’s correlation coefficients (r) and glmmLasso estimates.

**Table 3:** Comparative performance analysis of machine/deep learning algorithms for predicting responders/non-responders and remitters/non-remitters on testing data (VGHTP).

Algorithms	Responders/non-responders	Remitters/non-remitters
L1-regularied logistic regression	0.625 (0.541, 0.707)	0.768 (0.692,0.840)
Support vector machine (SVM)	0.605 (0.515, 0.688)	0.808 (0.727, 0.880)
Extreme gradient boosting (XGBoost)	0.570 (0.483, 0.658)	0.808 (0.716, 0.883)
Deep Neural network (DNN)	0.583 (0.496, 0.676)	**0.859 (0.795, 0.918)**
Ensemble	**0.631 (0.544, 0.710)**	0.816 (0.733, 0.882)

The performance is assessed using the area under the receiver operating characteristic curve (AUC), along with 95% confidence intervals. Bold values indicate the best performance in the comparisons.

When predicting remitters versus non-remitters, we added P.I. at week 4 as an extra predictor. Under these conditions, the support vector machine (SVM) algorithm attained the highest AUC of 0.859 (95% CI: 0.795, 0.918). Figure 1B illustrates how the predictive influence of the P.I. at week 4 exceeded that of other variables, underscoring the importance of incorporating current treatment response data to refine long-term SSRI outcome predictions.

**Fig. 2:**
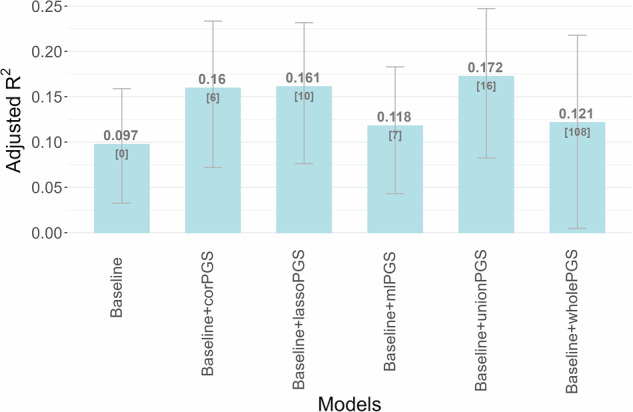
Adjusted R^2^ for linear models with the outcome as the percentage improvement in HRSD-17 scores (P.I.) at week 4, utilizing pooled data. The baseline model includes the following covariates: sex, age, age and sex interaction, number of depressive episodes, type and dosage of antidepressant drug, and HRSD-17 baseline score. We compared the adjusted R^2^ values with the inclusion of polygenic score (PGS) predictors selected through various approaches. These approaches involved PGS predictors demonstrating significant correlations with P.I. at week 4 (Baseline + corPGS), PGS predictors identified by glmmLasso (Baseline + lassoPGS), PGS predictors ranked among the top 10 features in the SHAP value selection (Baseline + mlPGS), as well as the union (Baseline + unionPGS) of PGS predictors from the three strategies. Additionally, the adjusted R-squared values are computed using all 108 PGS predictors together (Baseline + wholePGS). The number of PGS predictors integrated into each model is indicated in square brackets for reference. The error bars represent the 95% confidence intervals of the adjusted R^2^ values.

Lastly, Fig. 2 depicts the adjusted R^2^ values for linear models using pooled data, with the P.I. at week 4 as the dependent variable. Across all models, adding PGS predictors raised the adjusted R^2^ beyond that of the baseline model (adjusted R^2^ = 0.097), which included only demographic and clinical factors. In particular, the unionPGS model achieved the highest adjusted R^2^ value of 0.172, highlighting the enhanced explanatory power provided by these selected PGS predictors for SSRI treatment outcomes. AUC comparisons between the baseline model and models incorporating PGS predictors are presented in Supplementary Fig. S2 (Supplement Appendix 1).

## Discussion

In this study, we investigated the association between polygenic score (PGS) predictors for 108 distinct traits and SSRI outcomes in a Taiwanese population. To the best of our knowledge, this represents the first comprehensive exploration of how a wide range of PGSs might relate to SSRI efficacy in an East Asian population. We employed advanced methodologies, particularly PRS-CSx, to address the limited availability of East Asian GWAS data for generating PGSs. Our machine and deep learning algorithms demonstrated moderate predictive performance (AUC = 0.631) for distinguishing responders from non-responders, and notably, adding the percentage improvement in HRSD-17 scores at week 4 significantly enhanced predictive accuracy, achieving an AUC of 0.859 for identifying remitters versus non-remitters at week 8. This finding aligns with previous research that suggests early improvement may indicate later remission in antidepressant treatment [56]. Although we focused on week 4 due to data availability across both cohorts, future studies with harmonized measurements at earlier time points (e.g., week 1 or 2) are warranted. Such designs may enhance predictive accuracy and better capture individual variation in treatment response. Overall, our findings underscore both the promise of multi-PGS approaches in forecasting SSRI treatment outcomes and the importance of continually updating drug response research. The consistency of predictor patterns across percentage improvement, response, and remission underscores the utility of multi-PGS modeling even when continuous symptom change exhibits low heritability in previous studies [24].

Our analyses highlighted critical PGS predictors, most notably those related to insomnia, multisite chronic pain, and inflammatory biomarkers, which substantially influenced SSRI responses. Comorbidity of insomnia with MDD is well-established, with earlier studies [57–59] noting that certain SSRIs may alter sleep patterns through changes in rapid eye movement and induction of sleep disturbances. Bei et al. [60] found that insomnia severity correlates with poorer prognosis and reduced SSRI efficacy. Consistent with our findings, Lo et al. [61] also observed an association between the PGS for insomnia and non-remission rates in SSRI treatment, in data drawn from ten studies by the Psychiatric Genomics Consortium Major Depressive Disorder treatment response group, primarily of European ancestry. In relation to multisite chronic pain, our results indicate that a higher genetic predisposition to this condition is tied to diminished SSRI efficacy, echoing the conclusions of Campos et al. [62] in the Australian Genetics of Depression Study.

Additionally, our results imply that a natural predisposition to inflammation may be associated with a poor response to SSRI treatment. This is consistent with recent evidence that inflammation may contribute to pathology in certain patients with MDD [63, 64], and that SSRI treatment resistance in MDD is linked to elevated inflammatory markers [65, 66]. Mechanistically, pro-inflammatory cytokines can activate indoleamine-2,3-dioxygenase, redirecting tryptophan from serotonin synthesis toward neuroactive kynurenines, blunting the pharmacodynamic efficacy of SSRIs; they also dampen dopaminergic signaling and impair BDNF-mediated synaptic plasticity, further limiting clinical improvement [67–69]. These observations have led to the concept of an “inflamed-depression” subtype, in which elevated C-reactive protein or interleukin-6 predicts poorer SSRI response yet may identify patients who benefit from alternative agents or anti-inflammatory augmentation [70, 71]. Although adiposity is a recognized driver of low-grade inflammation, sensitivity analyses adjusting for PGS of BMI and obesity-related traits only partially attenuated the association between inflammatory PGSs and outcome, suggesting inflammation exerts effects beyond obesity-related severity. These convergent observations across diverse populations point to a complex interplay among genetic factors, insomnia, chronic pain, inflammation, and SSRI response, meriting further inquiry.

Our data show that the increment in explained variance (R² from 0.097 to 0.172) by adding PGSs for predicting P.I. at week 4 is relatively modest. It is likely that the clinical features in the baseline model already capture a substantial portion of the SNP-heritability for treatment outcomes, which limits the additional R² that can be gained by adding PGSs to the model. However, the improvement in predictive power gained by adding PGS should not be overlooked, as PGSs are fixed at birth, they can inform treatment-stratification decisions even when comorbid symptoms are transient, unmeasured, or yet to emerge, making them a complementary tool rather than a replacement for clinical assessment. Similarly, in our analysis predicting remission at week 8, we found that improvement at week 4 plays the most critical role, which limits the contribution of PGSs in predicting remission at week 8. However, we believe the importance of PGSs lies in the fact that they represent baseline, treatment-agnostic liabilities. Unlike week-4 improvement, which requires four weeks of medication, PGSs are available before treatment begins. They can thus inform drug selection or early augmentation decisions at the first visit, when no symptom-trajectory information yet exists. Their relatively small incremental AUC must be interpreted in that pre-treatment context.

Despite these insights, our study has several limitations. First, although certain PGS predictors reached nominal significance, none remained significant after multiple testing corrections. Moreover, in R² comparisons, wide confidence intervals indicated substantial uncertainty, and statistical evidence for the effect of PGS predictors remains inconclusive. This highlights the need for larger sample sizes to robustly identify predictive genetic markers, and future studies incorporating out-of-sample R² validation may provide stronger evidence of predictive utility. Second, although we used advanced methods (PRS-CS and PRS-CSx) to derive PGSs, further improvements in PGS estimates rely on larger GWAS datasets that are well-matched to the target population. Ongoing efforts to collect extensive biobank data—especially from individuals of non-European ancestries—are likely to bolster PGS accuracy and enhance clinical applicability. Third, predicting SSRI outcomes remains inherently challenging. Even our strongest machine/deep learning model achieved an AUC of 0.631 for predicting responders versus non-responders, a level still insufficient for clinical utility. Given the multifactorial nature of SSRI response, future studies should consider integrating additional biomarkers, such as brain imaging and omics data, to enrich the predictive landscape [72]. Fourth, both cohorts ended follow-up at week 8. Although this interval reflects common clinical practice, it may not capture later responders; trials extending to 12 or 16 weeks—especially in comorbid obsessive-compulsive disorder—are needed to characterize sustained SSRI effects and to validate our multi-PGS predictors over longer horizons. Fifth, we could not formally test mediation by insomnia, pain, or inflammatory biomarkers because these phenotypes were not systematically captured; future prospective studies should integrate these state measures to clarify whether PGS effects are fully or only partly channeled through comorbid conditions. However, the persistence of PGS signals after adjustment for baseline HAMD-17 underscores a potential trait-level contribution. Lastly, we focused on outcomes related specifically to SSRIs; broadening the scope to encompass other SSRIs would provide a more comprehensive picture of predictive models across diverse pharmacological interventions.

Looking ahead, larger sample sizes, richer datasets, integrated biomarkers, and the inclusion of various treatment options all offer considerable promise for advancing the predictive power of PGS in clinical settings. By pioneering the use of multi-polygenic scores in an East Asian population, our study underscores the need to expand genomic research across diverse ancestries and demonstrates the feasibility of integrating multiple PGS predictors for more robust outcome predictions. This approach not only broadens the scope of existing pharmacogenomic research but also paves the way for novel frameworks that combine genetic, clinical, and other biomarker data to deliver more personalized antidepressant regimens. Ultimately, refining and expanding these methods may reduce healthcare costs and alleviate the overall disease burden of depression, as more precise prediction models guide personalized treatment strategies and improve patient outcomes.

## Supplementary information

Supplementary tables

Supplementary information

## Data Availability

GWAS summary statistics utilized for PGS calculation are publicly accessible for download or can be obtained upon request, with detailed information presented in the Supplementary Table S2.
