# Supplementary material for: Integrating multi-polygenic scores for enhanced prediction of antidepressant treatment outcomes in an East Asian population
Source: Neuropsychopharmacology. 2025 Oct 28;51(2):422–9. doi: 10.1038/s41386-025-02269-y (PMC12708836; doi:10.1038/s41386-025-02269-y)
Supplement: Supplementary file 2 — Supplementary information [file 41386_2025_2269_MOESM2_ESM.docx]

**Supplement for “Integrating Multi-Polygenic Scores for Enhanced Prediction of Antidepressant Treatment Outcomes in an East Asian Population”**

Shu-Chin Lin^1,2^, Chiu-Ping Fang^1^, Chia-Lin Hsu^1^, An-Nie Chung^1,3^, Tzu-Ting Chen^1^, Kai-Hsiang Hsu^1^, Chueh-Chun Yeh^1^, Jingyi Zheng^4^, ChaoYu Liu^5^, Chi-Shin Wu^6,7^, Chia-Yen Chen^8^, Po-Hsiu Kuo^9^, Shih-Jen Tsai^10,11^, Yu-Li Liu^1^, Yen-Feng Lin^1,12,13*^

^1^Center for Neuropsychiatric Research, National Health Research Institutes, Miaoli, Taiwan

^2^Institute of Statistics and Data Science, National Taiwan University, Taipei, Taiwan

^3^Department of Psychiatry, Taipei City Psychiatric Center, Taipei City Hospital, Taipei, Taiwan

^4^Department of Mathematics and Statistics, Auburn University, Auburn, USA

^5^Department of Psychiatry, School of Medicine, Yale University, New Haven, USA

^6^National Center for Geriatrics and Welfare Research, National Health Research Institutes, Miaoli, Taiwan

^7^Department of Psychiatry, National Taiwan University Hospital Yunlin Branch, Yunlin, Taiwan

^8^Biogen, Cambridge, Massachusetts, USA

^9^Department of Public Health & Institute of Epidemiology and Preventive Medicine, College of Public Health, National Taiwan University, Taipei, Taiwan

^10^Department of Psychiatry, Taipei Veterans General Hospital, Taipei, Taiwan

^11^Division of Psychiatry, School of Medicine, National Yang Ming Chiao Tung University, Taipei, Taiwan

^12^Department of Public Health & Medical Humanities, School of Medicine, National Yang Ming Chiao Tung University, Taipei, Taiwan

^13^Institute of Behavioral Medicine, College of Medicine, National Cheng Kung University, Tainan, Taiwan

*** Correspondence:**Yen-Feng Lin
[yflin@nhri.edu.tw](mailto:yflin@nhri.edu.tw)

# Appendix 1. Supplementary Results

Table S1 provides association estimates derived from the linear mixed model (LMM) that illustrate the demographic and clinical predictors influencing the percentage improvement in HRSD-17 scores in response to antidepressant treatment. The extended treatment duration ("week") is positively associated with a more substantial improvement in HRSD-17 scores, a consistent trend observed across both cohort-specific and pooled analyses as depicted in Figure 1 in the main paper. Furthermore, the baseline HRSD-17 scores show a statistically significant positive correlation with the percentage improvement in HRSD-17 scores, suggesting that greater baseline severity may indicate better outcomes. Among the various medications, only escitalopram demonstrates a marginally significant improvement in HRSD-17 scores compared to the reference medication. Gender disparities are evident, with female patients, on average, displaying a 6.169% lower improvement compared to their male counterparts. An age and sex interaction effect is also observed, where older female patients exhibit enhanced treatment responses, while older male patients experience diminished responses. Specifically, in the pooled analysis, each additional year of age among male patients is associated with a 0.290% reduction in improvement at week 4, whereas older female patients see a 0.262% increase in improvement (the sum of sex and age×sex associations).

Figure S1 displays scatter plots and regression coefficient estimations obtained through simple linear regression, with separate estimations for males and females. In the VGHTP cohort, we observed that each additional year of age in males was associated with a reduction in the percentage improvement of HRSD-17 scores from baseline to week 4 by -0.33% (P-value=0.021), while each additional year of age in females was associated with an increase in the percentage improvement of HRSD-17 scores by 0.34% (P-value=0.010). In the NHRI cohort, the results are mainly consistent, with each additional year of age in males resulting in a reduction of percentage improvements in HRSD-17 scores by -0.23% (P-value=0.232), and each additional year of age in females yielding an increase of percentage improvements by 0.39% (P-value=0.003). Despite these significant findings, the influence of gender, age, and depression severity on antidepressant outcomes remains inconclusive (see Introduction of the main paper). Further study is required to determine if these findings are subject to population-specific effects.

Figure S2 presents. ROC analyses comparing model performance with and without polygenic score (PGS) predictors for the response at week 4. As shown in the figure below, the inclusion of PGS predictors resulted in a slight increase in discriminative performance, with the AUC rising from 0.605 (baseline model) to 0.631 (model with PGS predictors) using the ensemble model.

Table S1. Association estimates (standard errors) between demographic and clinical factors and the percentage improvement HRSD-17 scores in response to antidepressants based on the linear mixed model (LMM).

| **Variables** | **Class** | **Pooled Data** | **VGHTP** | **NHRI** |
| --- | --- | --- | --- | --- |
| **Week** | -- | 3.684***  (0.170) | 2.045***  (0.251) | 3.983***  (0.194) |
| **Sex** | Male | Reference |  |  |
|  | Female | -6.169**  (2.219) | -4.995  (3.057) | -5.186  (3.183) |
| **Age** | -- | -0.290*  (0.117) | -0.368*  (0.146) | -0.222  (0.188) |
| **Age**$\boldsymbol{\times}$**Sex** | -- | 0.552***  (0.138) | 0.625**  (0.191) | 0.506*  (0.211) |
| **Baseline HRSD-17 scores** | -- | 0.926***  (0.221) | 0.725*  (0.356) | 1.060***  (0.285) |
| **Number of depressive episodes** | -- | -0.972  (0.842) | 0.548  (1.606) | -1.528  (1.014) |
| **Drug** | Fluoxetine | Reference | Reference | -- |
|  | Citalopram | 2.320  (3.144) | 0.822  (5.037) | -- |
|  | Paroxetine | 2.320  (3.144) | -- | Reference |
|  | Escitalopram | 7.654*  (3.291) | -- | 4.986  (2.546) |
| **Dose of antidepressant drug** | -- | -2.510  (5.033) | -14.341  (18.385) | -1.412  (5.466) |

^*^p<0.05; ^**^p<0.01; ^***^p<0.001

#



$\beta$= 0.34 (0.13)

$\beta$= -0.33 (0.14)

$\beta$= 0.39 (0.13)

$\beta$= -0.23 (0.19)

Figure S1. Scatter plots and regression coefficients (standard errors) estimated by simple linear regression. The regression estimates are separately shown for males and females, illustrating the interaction between gender and age on the percentage improvements of HRSD-17 scores from baseline to week 4 for patients from the VGHTP and NHRI cohorts.


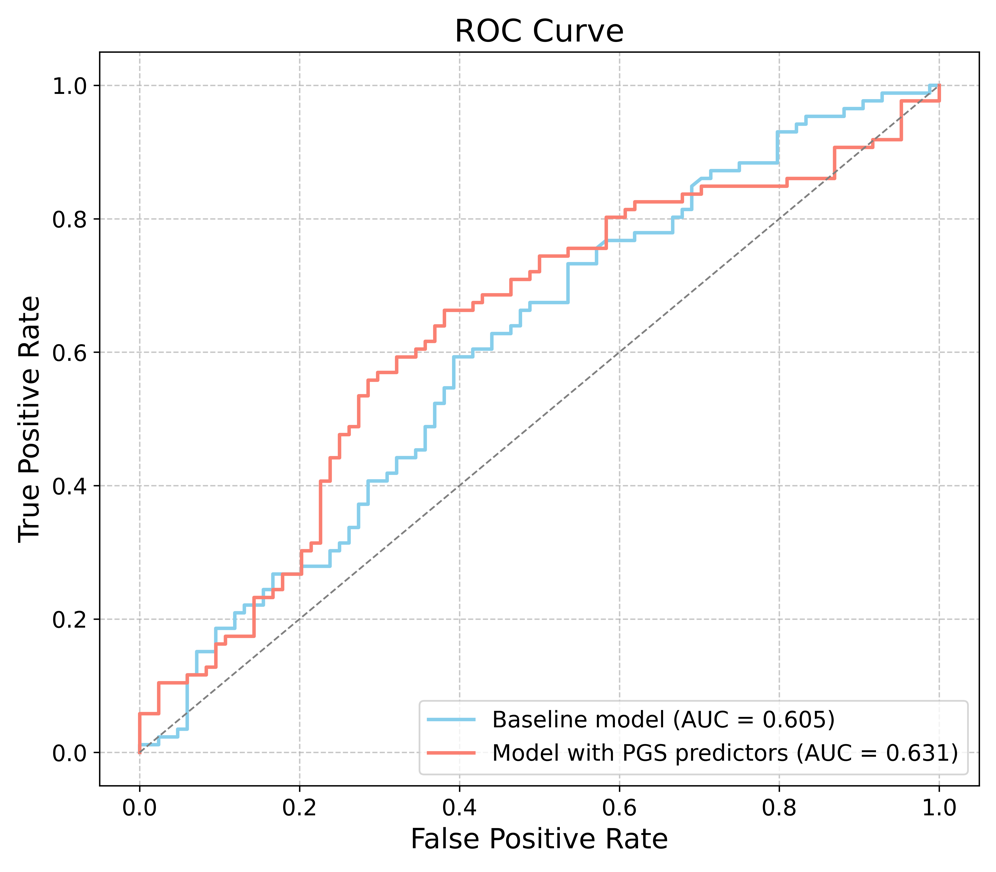


Figure S2. Receiver operating characteristic (ROC) curves for predicting remission using the baseline model (clinical and demographic variables only; blue line) and the model including polygenic score (PGS) predictors (red line). The AUC increased from 0.605 to 0.631 with the inclusion of PGSs, indicating a modest improvement in prediction performance.

# Appendix 2. Supplementary Methods

Figures S3 and S4 illustrate the workflows of our three main analyses: (1) Pearson correlation and (2) LMM with L1 regularization using continuous outcomes (percentage improvement), and (3) machine/deep learning analysis using the NHRI cohort for training and the VGHTP cohort for testing.

P.I. in HRSD-17

(week4)

NHRI (N=245)

&

VGHTP(N=174)

Pearson’s correlation analysis

PGSs

(108 traits)

P.I. in HRSD-17

(VGHTP: week 4,8)

(NHRI: week 1,2,4,6,8)

NHRI (N=245)

&

VGHTP(N=174)

Linear mixed model with L1-penalization

Baseline covariates

PGSs

(108 traits)

Figure S3. Pooled analysis of continuous outcomes: Analytical framework for estimating associations between percentage improvement (P.I.) in HRSD-17 scores and polygenic scores (PGSs) using Pearson’s correlation and linear mixed models with L1-penalization, based on combined NHRI and VGHTP cohorts.

Response / non-response

Or

Remission / non-remission

NHRI (N=245)

Training data

PGSs

(108 traits)

VGHTP(N=174)

Machine / Deep learning algorithm

Baseline covariates

Response / non-response

Or

Remission / non-remission

PGSs

(108 traits)

Baseline covariates

Trained model

Testing data

External validation:

AUC & Shapley value analysis

Figure S4. Machine learning analysis with training/testing design: Predictive modeling of response and remission using NHRI for training and VGHTP for external validation with baseline covariates and PGSs.

To build the prediction model for predicting responders/non-responders and remitters/non-remitters, we employed several machine learning algorithms, including L1-regularized logistic regression, support vector machine (SVM), and extreme gradient boosting (XGBoost), using the Scikit-learn library version 1.0.2 ([1](#_ENREF_1)). We used grid search strategies to determine optimal hyperparameters. Specifically, L1-regularized logistic regression was tuned by varying the parameter C over the values 10^{-6, -5, …, 1}^, using the 'liblinear' solver. Support vector machine (SVM) was optimized with the parameter C over the same range of values 10^{-6, -5, …, 1}^, employing the 'poly' kernel. For extreme gradient boosting (XGBoost), the following hyperparameters were explored: 'n_estimator' (500), 'learning_rate' (0.01), 'gamma' (values: 0, 1, 2, 3), 'colsample_bylevel' (values: 0.1, 0.3, 0.5, 0.7, 0.9), 'colsample_bynode' (values: 0.1, 0.3, 0.5, 0.7, 0.9), 'colsample_bytree' (values: 0.1, 0.3, 0.5, 0.7, 0.9), 'subsample' (values: 0.8, 1), 'min_child_weight' (values: 1,3,5,7,9), and 'max_depth' (values: 1, 2, 3, 4, 5).

For the deep neural network (DNN) **(**[2](#_ENREF_2)**)**, it was designed with two hidden layers. The first hidden layer consisted of 30 neurons, while the second layer comprised 10 neurons. Both layers utilized the rectified linear unit ('relu') activation function. Additionally, we incorporated dropout rates of 70% and 30% for the respective hidden layers. The network was concluded with a 'softmax' output layer, responsible for converting outputs into probability scores. During training, we used a batch size of 32, and a grid search was conducted for the number of epochs (values: 100, 200) and L2-regularized parameters (values: 0, 1, 2, 3, 4, 5, 6). An "epoch" refers to an iteration that involves adjusting the weights for all samples in the training dataset. The DNN learned classifier was implemented using Keras Tensorflow 2.11.0 in Python 3.9.12.

# References

1. Pedregosa F, Varoquaux G, Gramfort A, Michel V, Thirion B, Grisel O, et al. Scikit-learn: Machine learning in Python. the Journal of machine Learning research. 2011;12:2825-30.

2. Chollet F. Keras: The python deep learning library. Astrophysics source code library. 2018:ascl: 1806.022.
